# The reimbursement system can influence the treatment choice and favor joint replacement versus other less invasive solutions in patients affected by osteoarthritis

**DOI:** 10.1186/s40634-023-00699-5

**Published:** 2023-12-23

**Authors:** Luca De Marziani, Angelo Boffa, Alessandro Di Martino, Luca Andriolo, Davide Reale, Alessio Bernasconi, Valentina Rita Corbo, Francesca de Caro, Marco Delcogliano, Giorgio di Laura Frattura, Giovanni Di Vico, Andrea Fabio Manunta, Arcangelo Russo, Giuseppe Filardo

**Affiliations:** 1 Clinica Ortopedica e Traumatologica 2, IRCCS Istituto Ortopedico Rizzoli, Via Giulio Cesare Pupilli, Bologna, 1 - 40136 Italy; 2 Ortopedia e Traumatologia, IRCCS Istituto Ortopedico Rizzoli, Bologna, Italy; 3 Orthopaedics and Traumatology Unit, Department of Public Health, University Federico II of Naples Federico II, Naples, Italy; 4 Foot and Ankle Specialistic Unit, IRCCS Galeazzi Sant’Ambrogio, Milan, Italy; 5 Department of Orthopaedic Surgery, Istituto Di Cura Città Di Pavia, Pavia, Italy; 6 Servizio di Ortopedia e Traumatologia dell’Ospedale Regionale di Bellinzona e Valli, Ente Ospedaliero Cantonale, Ticino, Switzerland; 7 Division of Orthopedics and Trauma Surgery, Geneva University Hospitals, Geneva, Switzerland; 8 Department of Orthopaedics and Trauma Surgery, Clinica San Michele, Maddaloni, Italy; 9 UOC di Clinica Ortopedica AOU Sassari, Sassari, Italy; 10 Humanitas Castelli, Bergamo, Italy; 11 Applied and Translational Research (ATR) Center, IRCCS Istituto Ortopedico Rizzoli, Bologna, Italy; 12 Service of Orthopaedics and Traumatology, Department of Surgery, EOC, Lugano, Switzerland; 13 Faculty of Biomedical Sciences, Università della Svizzera Italiana, Lugano, Switzerland

**Keywords:** Osteoarthritis, Prosthesis, Arthroplasty, Diagnosis-Related Groups, DRG, Reimbursement

## Abstract

**Purpose:**

The aim of this study was to assess how physicians perceive the role of the reimbursement system and its potential influence in affecting their treatment choice in the management of patients affected by osteoarthritis (OA).

**Methods:**

A survey was administered to 283 members of SIAGASCOT (Italian Society of Arthroscopy, Knee, Upper Limb, Sport, Cartilage and Orthopaedic Technologies), a National scientific orthopaedic society. The survey presented multiple choice questions on the access allowed by the current Diagnosis-Related Groups (DRG) system to all necessary options to treat patients affected by OA and on the influence toward prosthetic solutions versus other less invasive options.

**Results:**

Almost 70% of the participants consider that the current DRG system does not allow access to all necessary options to best treat patients affected by OA. More than half of the participants thought that the current DRG system favors the choice of prosthetic solutions (55%) and that it can contribute to the increase in prosthetic implantation at the expense of less invasive solutions (54%). The sub-analyses based on different age groups, professional roles, and places of work allowed to evaluate the response in each specific category, confirming the findings for all investigated aspects.

**Conclusions:**

This survey documented that the majority of physicians consider that the reimbursement system can influence the treatment choice when managing OA patients. The current DRG system was perceived as unbalanced in favor of the choice of the prosthetic solution, which could contribute to the increase in prosthetic implantation at the expense of other less invasive options for OA management.

## Introduction

Osteoarthritis (OA) represents an important public health problem and one of the world’s leading disabling diseases [19]. Its prevalence has been steadily rising over the years, due to demographic shifts such as aging population, an upsurge in overweight individuals, and a more physically active lifestyle among the elderly population [50]. This led to a significant increase in the number of total joint replacement surgeries, which represent the end-stage treatment for OA patients. Total joint arthroplasty (TJA) is considered one of the most successful surgical innovations of the 20th century, thanks to its proved efficacy in relieving pain and improving function in a durable and cost-effective manner when appropriately indicated [1, 40, 63]. The volume of these procedures has risen dramatically over the past several decades, with over 800,000 total hip and knee arthroplasties being performed annually in the United States only [17, 24, 32, 72]. Moreover, a further increase is expected with 1.22 million prostheses foreseen by 2040 in the United States [72]. This implies important consequences for the healthcare systems [61, 72].

The growth in TJA can be viewed as a consequence of the success of these procedures, with an expansion of indications and the tendency of surgeons to perform joint replacement also in younger patients and in earlier OA stages [18, 44, 55, 73]. Almost a third of primary total knee arthroplasties (TKAs) is performed in patients younger than 65 years, where the indication of prosthesis is raising, with patients aged between 45 and 55 years being the fastest increasing age group [17, 34, 37, 41, 55, 78]. However, results in younger patients are less satisfactory, and they present a higher risk of needing revision surgery in their lifetime [67, 77]. In this light, it would be better to bring an increasing number of patients up to an age where their life expectancy matches the longevity of joint replacement [68]. To this end, several alternative less invasive approaches have been developed with promising results, ranging from the more documented treatments like intra-articular injections and osteotomies [59], to new experimental options like subchondral bone injections, intra-articular spacers, and implantable shock absorbers [35, 51, 71]. Still, the available strategies to postpone metal resurfacing do not seem able to contrast the increase in prosthetic replacements. A possible explanation for this trend in treatment indications could be related to the reimbursement system. In fact, procedures are currently associated with Diagnosis-Related Groups (DRG), each one corresponding to a specific value that is reimbursed by the health system [13]. Reimbursements differ among the different treatment strategies, and this could influence the medical choice [16, 33].

The aim of this study was to assess how physicians perceive the role of the reimbursement system and its potential influence in affecting their treatment choice in the management of OA patients.

## Material and methods

A survey was prepared by the members of the Cartilage and Regenerative Medicine Committee of SIAGASCOT (Italian Society of Arthroscopy, Knee, Upper Limb, Sport, Cartilage and Orthopaedic Technologies), a National scientific orthopaedic society. The survey was a self-administered questionnaire in Italian language that was first distributed among society members at the Society’s National congress in March 2022. To further increase the response rate the survey was then administered at the faculty meeting (SIAGASCOT Day) in October 2022. The survey presented three multiple choice questions, and each question was kept short, simple, and unambiguous to specifically answer the study objectives. The survey also asked supplementary questions to capture demographic information on the respondents, including age, professional role, and place of work (Table 1). The questionnaire was completed anonymously and not traceable to individual participants. The inclusion criteria for this survey included all physicians attending the SIAGASCOT events. Data obtained from the completed questionnaires were transferred in a spreadsheet and then analyzed using Microsoft Excel (Microsoft Office 365 for Windows). Incomplete surveys were eliminated from the analysis and data have been reported per-protocol. Possibly duplicated answers were sorted based on demographic and professional data and eliminated. Results were presented as frequencies and percentages, following the guidelines “Guidelines for Reporting Survey-Based Research Submitted to Academic Medicine” [5].

**Table 1:** Schematic representation of the survey

**N.**	**Question**	**Answers**
**1**	“How old are you?”	□ < 30 □ 30–40 □ 40–50 □ 50–60 □ > 60
**2**	“Which is your professional role?”	□ Orthopaedic resident □ Non-orthopaedic resident □ Orthopaedic specialist □ Non-orthopaedic specialist □ Director of orthopaedic clinic □ Director of non-orthopaedic clinic
**3**	“Which is your place of work?”	□ National Health Service (NHS) □ Private accredited to NHS □ Private practice
**4**	“Do you think that the current DRG system allows access to all necessary options to best treat patients affected by OA?”	□ Yes □ No □ I do not know
**5**	“Do you think that DRGs for OA treatments are unbalanced and favor the choice of prosthetic solutions?”	□ Yes □ No □ I do not know
**6**	“Do you think that the current DRG system can contribute to the increase in prosthetic implantation at the expense of other less invasive solutions?”	□ Yes □ No □ I do not know

## Results

**Fig. 1:**
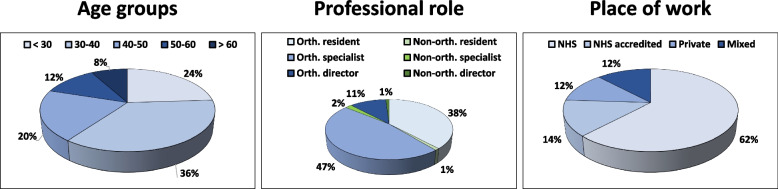
Characteristics of the responders to the survey

A total of 295 members of the SIAGASCOT society completed the survey. Among the completed questionnaires, 283 were included in the analysis, while 12 were excluded because they were completed by non-physician members (researchers, physiotherapists, osteopaths). In detail, 139 were specialists (132 orthopaedic and 7 not-orthopaedic), 109 were residents (106 orthopaedic and 3 not), and 35 were directors of operating units (31 orthopaedic and 4 not). The most represented age was 30–40 years (36.4%), followed by < 30 years (23.7%), 40–50 years (19.8%), 50–60 years (12.3%), and > 60 years (7.8%). Regarding the place of work, over half of the participants (61.8%) were working in the National Health Service (NHS), 14.1% in private clinics accredited to the NHS, 12.0% in the private practice, while the remaining 12.1% in other frameworks (i.e., physicians working in both NHS and private practice). A schematic representation of the demographic characteristics of the responders is reported in Table 2 and Fig. 1, while a schematic representation of the responses to the survey questions is reported in Table 3. A more detailed representation and analysis of the responses is presented in the following paragraphs.

**Table 2:** Demographic characteristics of the responders to the survey

**Questions**	**Options**	**Responses**	**Percentage**
***“How old are you?”***	< 30	67	23.7%
	30–40	103	36.4%
	40–50	56	19.8%
	50–60	35	12.3%
	> 60	22	7.8%
***“Which is your professional role?”***	Orthopaedic resident	106	37.5%
	Non-orthopaedic resident	3	1.0%
	Orthopaedic specialist	132	46.6%
	Non-orthopaedic specialist	7	2.5%
	Director of orthopaedic clinic	31	11.0%
	Director of non-orthopaedic clinic	4	1.4%
***“Which is your place of work?”***	NHS	175	61.8%
	Private NHS accredited	40	14.1%
	Private practice	34	12.0%
	NHS + NHS accredited	4	1.4%
	NHS + Private practice	5	1.8%
	NHS accredited + Private practice	23	8.2%
	NHS + NHS accredited + Private practice	2	0.7%

**Table 3:** Responses to the survey

**Questions**	**Options**	**Responses**	**Percentage**
***Question 1*** *“Do you think that the current DRG system allows access to all necessary options to best treat patients affected by OA?”*	Yes	28	9.9%
	No	196	69.3%
	I do not know	59	20.8%
***Question 2*** *“Do you think that DRGs for OA treatments are unbalanced and favor the choice of prosthetic solutions?”*	Yes	157	55.5%
	No	61	21.5%
	I do not know	65	23.0%
***Question 3*** *“Do you think that the current DRG system can contribute to the increase in prosthetic implantation at the expense of other less invasive solutions?”*	Yes	153	54.1%
	No	72	25.4%
	I do not know	58	20.5%

### “Do you think that the current DRG system allows access to all necessary options to best treat patients affected by OA?”

**Fig. 2:**
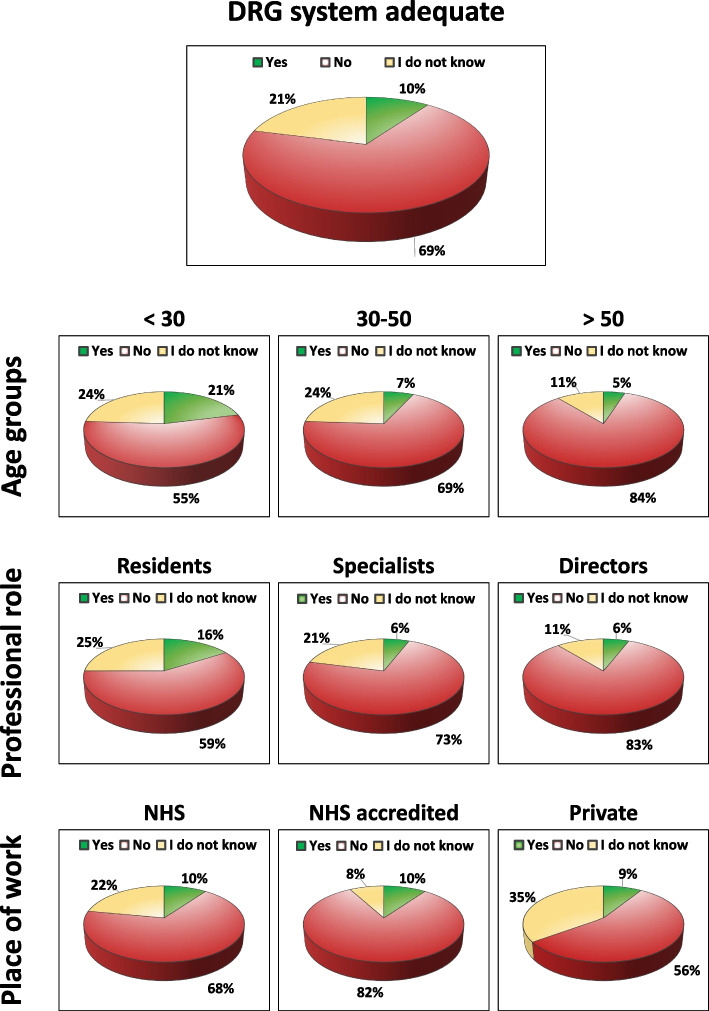
Answers to the question on the adequacy of the current DRG system to best treat patients affected by OA

Almost 70% of the participants thought that the current DRG system does not allow access to all necessary options to best treat patients affected by OA, 20% responded “I do not know”, while only 10% considered adequate the current DRG system for OA treatment. Analyzing the answers based on age groups, a trend has been observed: the current DRG system has been considered inadequate by 82% of over 60 physicians, by 86% of the group 50–60 years old, by 73% of the group 40–50 years old, by 67% of the group 30–40 years old, and by 55% of the group under 30. Excluding the residents from the analysis, the response trend was confirmed also for the specialists alone (including directors of clinic) with 75% considering the current DRG system inadequate. Regarding the place of work, the current DRG system has been considered not adequate by 82% of physicians working in the private NHS accredited, by 71% of physicians working in other frameworks, by 68% of physicians working in the NHS, and by the 56% of physicians working in private practice. More details on this question are reported in Fig. 2.

### “Do you think that DRGs for OA treatments are unbalanced and favor the choice of prosthetic solutions?”

**Fig. 3:**
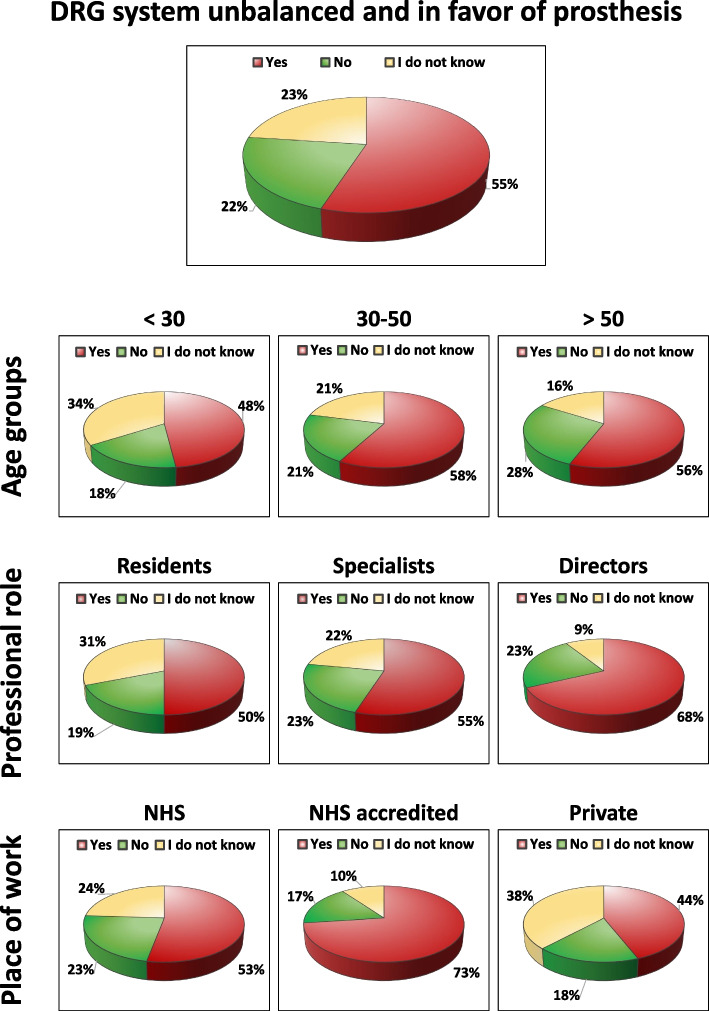
Answers to the question on the current DRG system being unbalanced towards the choice of prosthetic solutions

More than half (55%) of the participants thought that the current DRG system is unbalanced and favors the choice of prosthetic solutions, 23% responded “I do not know”, while only 22% considered balanced the current DRG system for OA treatment. Based on age groups, the current DRG system has been considered unbalanced by 50% of physicians over 60, by 60% of the group 50–60 years old, by 64% of the group 40–50 years old, by 54% of the group 30–40 years old, and by 48% of the group under 30. Excluding the residents from the analysis, the response trend was confirmed also for the specialists alone (including directors of clinic) with 58% responding that the current DRG system is unbalanced and favors the choice of prosthetic solutions. Regarding the place of work, the current DRG system has been considered unbalanced and in favor of the choice of prosthetic solutions by 73% of physicians working in the private NHS accredited, by 60% of physicians working in other frameworks, by 53% of physicians working in the NHS, and by 44% of physicians working in private practice. More details on the first question are reported in Fig. 3.

### “Do you think that the current DRG system can contribute to the increase in prosthetic implantation at the expense of other less invasive solutions?”

**Fig. 4:**
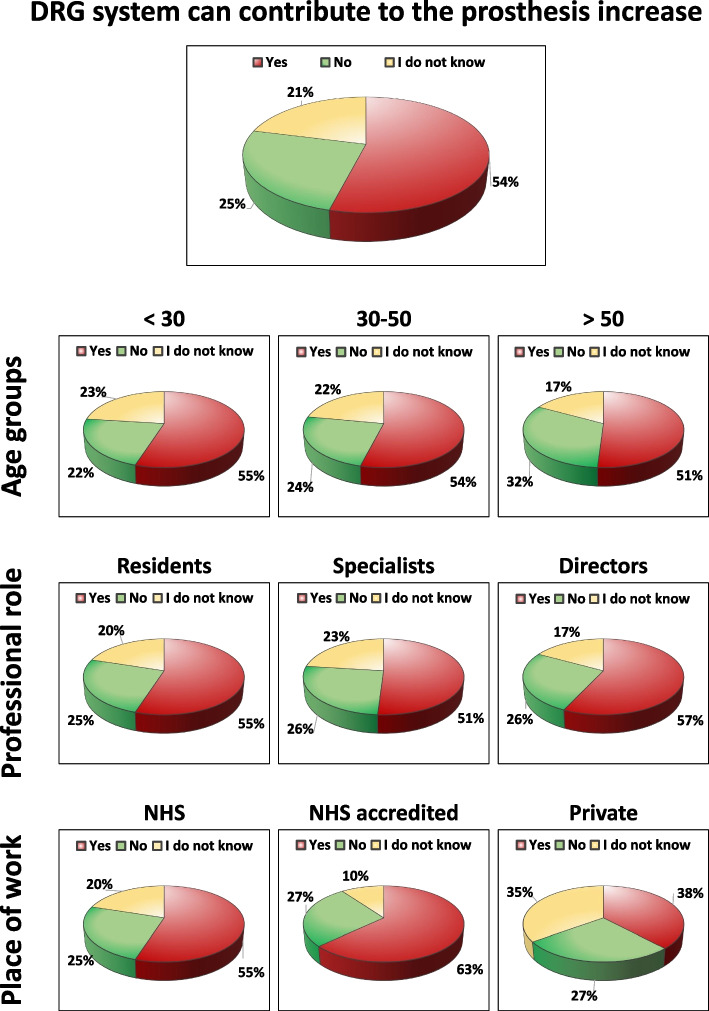
Answers to the question on the contribution of the current DRG system to the prosthesis implantation increase

More than half (54%) of the participants thought that the current DRG system can contribute to the increase in prosthetic implantation at the expense of other less invasive solutions, 21% responded “I do not know”, while only 25% of responders did not attribute the increase in prosthetic implantation to the DRG system. Based on age groups, the current DRG system has been considered to contribute to the increase in prosthetic implantation by 45% of physicians over 60, by 54% of the group 50–60 years old, by 61% of the group 40–50 years old, by the 50% of the group 30–40 years old, and by 55% of the group under 30. Excluding the residents from the analysis, the response trend was confirmed also for the specialists alone (including directors of clinic) with 52% responding that the current DRG system can contribute to the increase in prosthetic implantation. Regarding the place of work, the current DRG system has been considered to contribute to the increase in prosthetic implantation and in favor of the choice of prosthetic solutions by 63% of physicians working in the private NHS accredited, by 47% of physicians working in other frameworks, by 55% of physicians working in the NHS, and by 38% of physicians working in private practice. Other details on this question are reported in Fig. 4.

## Discussion

The main finding of this survey is that physicians consider the current DRG system inadequate and unbalanced in favor of the choice of the prosthetic solution. This seems to contribute to the increase in prosthetic implantation at the expense of other less invasive options for OA management. Overall, orthopaedic surgeons consider that the reimbursement system affects their choice in the treatment of patients affected by OA.

The reimbursement system is an essential element in the public healthcare context, as it ensures access to medical care for patients with health issues while managing the available resources [39]. The DRG has become an important classification system in the global healthcare frameworks, including Europe, America, Asia, and Australia [54, 58]. Under the DRG system, hospitals are reimbursed a fixed amount based on the patient’s diagnosis and medical procedures, rather than on the actual costs incurred. While this approach offers cost predictability and standardization, it could also create financial pressures for medical facilities, especially when dealing with complex and expensive treatments, such as joint arthroplasty [7, 14, 46, 57]. Previous research suggested that the reimbursement system may influence physicians’ decisions in other fields [8, 31, 47], and this could also hold true for the therapeutic decision-making process of OA. In fact, reimbursements related to joint replacement are higher compared to those for conservative and less invasive options for the management of OA patients, which could lead to a potential preference among healthcare professionals toward the joint replacement option, as demonstrated by this survey.

This could have significantly contributed to the rise in the number of arthroplasty procedures performed in recent years, which goes beyond the increasing prevalence of OA over the years [30, 42]. The current survey confirmed that among orthopaedic surgeons there is a perception of inappropriate utilization of prosthetic implants at the expense of less invasive solutions. Joint arthroplasty should represent the definitive treatment for end-stage OA in patients who no longer respond to conservative and less-invasive procedures [25]. Nevertheless, recent registry analyses underlined that joint arthroplasty indications have been expanded to include patients with less severe forms of OA and at younger age [55, 73]. Large evidence suggested a less favorable outcome of joint replacement in this patient group [17, 34, 37, 41, 55].

It is crucial to balance the potential benefits of an improvement in their quality of life against the potential risk of poor functional outcomes and even more a not negligible risk of revision. A survival analysis on over 100,000 patients undergone total hip or knee replacement investigated the lifetime risks of revision surgery based on increasing age at the time of primary surgery [6]. It has been proven that for patients who are younger than 60 years at primary surgery, their lifetime risk of revision at 20 years increases significantly, reaching up to one in three in those patients aged 50–55 years. In contrast, older patients undergoing hip or knee replacement at or over 70 years of age had a lifetime risk of requiring revision surgery between 1 and 6%. Similarly, a recent study analyzed a regional registry involving 45,488 total knee replacements, finding that at 15 years of follow-up patients under 65 years old had a double risk of implant failure compared to older patients [55].

More than 200,000 revision surgeries are expected in the United States alone in 2030 [66]. Beside the important clinical implications to the affected patients, the costs of revision surgeries are a significant concern for the healthcare system, including the removal of the previous prosthesis, the implantation of a new prosthesis, hospital costs, and post-operative rehabilitation [52, 53]. The complexity of the surgery increases operating room time and hospital stay and entails higher complication rates compared to primary surgeries. Moreover, the removal of the previous prosthesis can lead to substantial loss of bone and soft tissues, making the implantation of a new prosthesis more challenging [4, 12, 65, 70]. There is an increase in the risk of infections, joint instability, misalignment, prosthesis loosening, as well as vascular and neurological complications [23, 60, 62]. Furthermore, patients undergoing revision may experience worsening of pre-existing clinical conditions, increased blood loss during surgery, and a longer and more complex post-operative recovery, further increasing the total costs [38, 49]. Overall, this can cause significant financial pressure on the healthcare system and could result in a negative impact on the resources and quality of the healthcare [57].

These findings are particularly important considering on one side the increasing life expectancy and on the other side the emergence of new joint preserving strategies, which could postpone the need for joint replacement to an older age. Several alternative solutions have been proposed to manage younger patients with early to moderate OA preserving the native joint, reducing the progression of joint damage and delaying the need for joint replacement. Among these, knee osteotomy is an established surgical treatment for young patients with mono-compartmental knee OA and lower limb misalignment [26, 29, 43]. Restoring the correct alignment of the lower limb reduces the overload on the affected compartment improving pain and function, slowing the deterioration of the knee, and delaying or avoiding the need for arthroplasty [9, 48]. A recent case-control study demonstrated a reduced need for prosthetic revision in young patients treated with high tibial osteotomy and then with TKA compared to young patients treated with an early TKA [22]. Therefore, knee osteotomy should be considered as a treatment option to postpone TKA in these patients. Restoring proper lower limb alignment reduces overload on the affected compartment, improving pain, function, and potentially delaying or avoiding the need for arthroplasty [26]. Similarly, some cartilage restoration procedures can represent a solution in young patients with focal chondral or osteochondral lesions in early OA joints [3, 11, 20, 36, 69], with good results reported at mid-term follow-up even for moderate stages of unicompartmental OA addressed with a combined approach of knee osteotomy and cartilage and meniscus scaffold/allograft implantations [15, 45].

Injective solutions show promise to target the whole joint environment, improving joint homeostasis and thus reducing symptoms and delaying the need for arthroplasty. Among the injective options, viscosupplementation is one of the most used in the clinical practice [21, 56], although there is no consensus among different scientific societies on guidelines for its use [10]. The repetitive use of hyaluronic acid has been suggested to postpone joint replacement in OA patients, with several retrospective analyses reporting a higher median time to TKA in patients who received hyaluronic acid injections compared to control groups [2, 75, 76]. Similarly, it has been demonstrated that also intra-articular platelet-rich plasma (PRP) injections are able to delay the need for joint arthroplasty, with a survival analysis on over 1,000 patients with knee OA reporting a median delay of 4 years and a survival rate of 85% at 5 years of follow-up [64]. Considering the lower invasiveness, the safety, and the promising results, these injectable options could be considered in the decision-making process for the management of patients with OA, in order to delay prosthesis. Currently, the reimbursement system does not favor the use of these minimally invasive solutions over total joint replacement, even in young patients with early OA.

Moreover, new applications of orthobiologics are emerging to target the subchondral bone, which is believed to play a role in the pathophysiology and progression of OA disease [74]. Subchondral bone marrow aspirate concentrate (BMAC) injections reported promising results in delaying TKA. In a randomized trial on 30 young patients with bilateral knee OA secondary to osteonecrosis, comparable clinical results up to 12 years have been observed in knees treated either with TKA or a subchondral BMAC injection, but with a lower complication rate and a quicker recovery for knees treated with the injective approach [27]. In a randomized trial on 140 adult patients with bilateral medial knee OA patients were randomly treated with subchondral BMAC injections on one side and TKA on the other side. The injective procedure provided an effect on pain sufficient to postpone or avoid TKA up to 15 years of follow-up, with only 25 patients requesting TKA in joints treated with BMAC injections [28]. Further research should confirm, consolidate, and optimize the promising findings of these studies, as well as investigate new minimally invasive solutions able to delay or avoid prosthetic joint replacement. Efficient alternatives are highly needed to be adopted by the healthcare systems to balance the pressure documented toward prosthetic replacement.

This study has some limitations. The number of participants was a subgroup of all society members, and the sample was not homogeneous in terms of age, professional role, and place of work. In particular, the inclusion of staff with few years of experience may have caused a bias related to the complete understanding of the reimbursement system currently in place. Nevertheless, the heterogeneity allowed the overall group of being representative of the entire physician population, and the sub-analyses based on different age groups, professional role, and place of work allowed to evaluate the response rate in each specific category. Moreover, the analysis performed excluding the youngest resident physicians confirmed the findings for all investigated aspects. Finally, due to the nature of the survey administered to members of a National society, results may be representative of the specific reimbursement system and the conclusions should be generalized with caution to other countries. On the other hand, the DRG systems present similarities in several countries across different continents, with a strong focus on the prosthetic solution which should be investigated critically according to the insights of this survey. The healthcare framework can drive the orthopaedic surgeon’s choice, which warrants caution towards providing a balanced reimbursement system offering the choice of prosthetic replacement not at the expense of less invasive and potentially more suitable solutions for the management of OA patients.

## Conclusions

This survey documented that the majority of physicians consider that the reimbursement system can influence the treatment choice when managing OA patients. The current DRG system was perceived as unbalanced in favor of the choice of the prosthetic solution, which could contribute to the increase in prosthetic implantation at the expense of other less invasive options for OA management.

## Data Availability

Not applicable.
